# Editorial: Bioactive compounds, functional ingredients, antioxidants, and health benefits of edible plants

**DOI:** 10.3389/fpls.2024.1420069

**Published:** 2024-05-21

**Authors:** Eman A. Mahmoud, Hosam O. Elansary

**Affiliations:** ^1^ Department of Food Science, Faculty of Agriculture, Damietta University, Damietta, Egypt; ^2^ Department of Plant Production, College of Food & Agriculture Sciences, King Saud University, Riyadh, Saudi Arabia

**Keywords:** natural products, antioxidants, biological activities, flavonoids, edible plants

Edible plants are rich in bioactive compounds that have physiological effects such as anticancer, antioxidant, anti-inflammatory, and antimicrobial activities. Natural plant extracts are frequently used to prolong the shelf life of fresh and processed foods, thereby preserving their quality and safety. Phytochemical studies of extracts and the biological activities of various plant organs are also important in the food and human nutrition industries. They have the potential to pave the way for the commercialization of other plants by developing new applications for the food sector. Plant bioactive compounds represent a promising research objective for plant breeders, producers, and food processing industries. The study of the relationship between health and food has increased steadily and exponentially in recent years. The variety of bioactive compounds contained in edible plants, in addition to the different mechanisms of action involved in human nutrition, make this subject widely debated and the focus of this Research Topic. Furthermore, recent advances in extraction techniques, analytical approaches, and bioactivity assays have allowed scientists to explore minor dietary components and metabolites with high bioactivity and their pathways. The development of novel technological approaches for the manufacturing and processing of edible plants is of great interest to the agricultural and food industries worldwide, whose objectives include increasing the production of secondary metabolites (e.g., polyphenols) and developing novel applications. The use of bioreactors in tissue culture is strongly associated with the production of specific secondary metabolites that have important applications.

The work of Yang et al. explored the regulatory mechanisms of flavonoid synthesis in *Populus × euramericana* ‘Zhonghuahongye’ by analyzing flavonoids, polyphenols, and anthocyanins in the red and green leaves. The researchers identified 273 flavonoid metabolites (114 flavones, 41 flavonols, 34 flavonoids, 25 flavanones, 21 anthocyanins, 18 polyphenols, 15 isoflavones, and 5 proanthocyanidins). Higher levels of flavonoids were found in the red leaves and specific flavonoids were responsible for the red color of the leaves. The coloration of the leaves was strongly associated with the *CHS*, *FLS*, *ANS*, and *LAR* genes. Biru et al. have conducted a comprehensive investigation on the effects of Si supplementation and herbivory by *Helicoverpa armigera* on the activities of antioxidant enzymes (CAT, SOD, and APX) in *Festuca arundinacea* under reduced and Anthropocene levels of CO2. They revealed that increasing CO_2_ concentrations have positive effects on plant mass and foliar carbon. The researchers also found a correlation between Si supplementation and the activity of APX and SOD enzymes in plants grown under different CO_2_ regimes. They suggested that *Festuca arundinacea* may be more susceptible to herbivory when subjected to specific CO_2_ concentrations.

Irchad et al. found that lipidomic profiling of *Ficus carica* L. (Fig.) seed cultivars can reveal phenotypic diversity and associated nutritional benefits. The study used FTIR-ATR spectroscopy and chemometric techniques to investigate 22 fig genotypes and to explore their nutritional properties (antioxidant properties, nutritious source of lipids, minerals, and proteins), genetic relationships (Lipochemical-based fingerprinting), and potential applications as a genetic pool for future breeding programs for oil production. The findings of this study are highly relevant to the food industry. Cerulli et al. studied the metabolomics of lipids and phenols in hazelnut (*Corylus avellana* L., Betulaceae) of *n*-butanol extracts by LC-ESI/HRMS in the polar fraction of fresh and roasted kernels. The radical scavenging activity of the isolated compounds was determined using TEAC and TBARS assays. They identified phenolic compounds, polar lipids, flavonoids, and diarylheptanoid derivatives. The majority of the identified compounds showed strong antioxidant activities. The results of this are also highly relevant to the food industry. Zhang et al. conducted a comparative study on two pomegranate (*Punica granatum* L.) cultivars, ‘Tunisia’ and ‘Qingpi’, to elucidate the phenolic composition and antioxidant activities in different plant parts including fruits, flowers, and leaves. They revealed significant differences in the phenolic composition of plant organs, with petals showing the highest polyphenol and total anthocyanin compositions. The flavonoids and punicalagin were at the highest levels in the placenta, while the flavanols were at the highest levels in the peel. The highest antioxidant activity was found in fruits, followed by flowers and leaves; and was positively associated with total polyphenols. The study revealed that the placenta is the main source of punicalagin. The results of this are highly relevant to the pharmaceutical and food industries. Xu et al. revealed the nutritional value, antioxidant activity, and volatile compounds in three cultivars of jaboticaba berry (Sabara, Argentina, and Fukuoka). The authors found that the Sabara cultivar was rich in volatiles, had a suitable acid-sugar ratio, had low antioxidant capacity, and was recommended for fresh consumption. The Argentina cultivar had the most volatile compounds and was suitable for dry products. Finally, the Fukuoka cultivar had the largest fruit, with juicy flesh, and was suitable for juice processing. Similarly, Raza et al. studied different tomato (*Solanum lycopersicum* L.) genotypes to reveal the diversity in fruit nutritional composition, antioxidant, and biochemical profiles. They found that specific cultivars were rich in chlorophylls, lycopene, carotenoids, total antioxidant capacity, protease, alpha-amylase, and flavonoids, while other cultivars were rich in total soluble sugars, reducing sugars, malondialdehyde, ascorbic acid, esterase, peroxidase, and superoxide dismutase.

Abidizadegan et al. in their work presented an in-depth analysis of the impact of iron application on phycoerythrin, extracellular polymeric substances, and phenolic compounds isolated from *C. pyrenoidifera* and *Cryptomonas* sp. and their antioxidant activities. The application of iron increased the growth rates of the cryptophytes, and influenced the composition of phycoerythrin and phenolic compounds and consequently the antioxidant activities. Liu et al. investigated the anti-inflammatory compounds of *Capparis spinosa* fruits using metabolome and transcriptome analyses. They found strong anti-inflammatory effects in the fruits and identified 15 compounds exhibiting anti-inflammatory activity, with phenolic compounds being the major anti-inflammatory components in *C. spinosa*. Jiang et al. studied the chemical composition and aroma changes during the processing of Jujube tea using LC-MS-based metabolomics and GC-IMS analysis. They identified 468 non-volatile metabolites and 52 and 24 volatile metabolites. They found that lipids and lipid-like molecules, organic acids, amino acids, and flavonoids increased in composition following processing. Wang et al. found that the variability in the bioactive compound composition of *Swertia mussotii* in wild distant geographical populations of China is associated with environmental conditions including altitude, aspect, soil TK content, Fe content, and C/N and N/P ratios. Finally, Liu et al. revealed the mechanism of action of the traditional Chinese medicine YU-Pingfeng San in alleviating inflammatory allergic rhinitis. They identified 30 active ingredients in three effective herbs associated with the treatment of symptoms of nasal congestion and allergic rhinitis.

## Author contributions

EM: Conceptualization, Data curation, Formal analysis, Funding acquisition, Investigation, Methodology, Project administration, Resources, Software, Supervision, Validation, Visualization, Writing – original draft, Writing – review & editing. HE: Conceptualization, Data curation, Formal analysis, Funding acquisition, Investigation, Methodology, Project administration, Resources, Software, Supervision, Validation, Visualization, Writing – original draft, Writing – review & editing.

